# Ultrasound assessment of skin thickness and stiffness: the correlation with histology and clinical score in systemic sclerosis

**DOI:** 10.1186/s13075-020-02285-x

**Published:** 2020-08-26

**Authors:** Chen Chen, Yi Cheng, Xiaoxia Zhu, Yehua Cai, Yu Xue, Ning Kong, Yiyun Yu, Dandan Xuan, Shucong Zheng, Xue Yang, Zaihua Zhu, Tianyi Zhao, Weiguo Wan, Hejian Zou, Minrui Liang

**Affiliations:** 1 Division of Rheumatology, Huashan Hospital, Fudan University, 12 Wulumuqi Zhong Road, Shanghai, 200040 China; 2 Institute of Rheumatology, Immunology and Allergy, Fudan University, Shanghai, China; 3 Department of Ultrasound, Huashan Hospital, Fudan University, Shanghai, China

**Keywords:** Ultrasound, Shear wave elastography, Systemic sclerosis, Histology

## Abstract

**Background:**

Ultrasound is a useful tool to evaluate and quantify skin lesions. Few studies have assessed the criterion validity of skin ultrasound in systemic sclerosis (SSc). The aims of the study were to investigate skin thickness and stiffness using ultrasound and shear wave elastography (SWE) in SSc and to validate skin ultrasound measurements against histological skin thickness.

**Methods:**

A total of 22 patients with diffuse cutaneous SSc (dcSSc), 22 with limited cutaneous SSc (lcSSc), and 22 age- and gender-matched healthy controls were enrolled. Skin thickness and stiffness were measured by B-mode ultrasound with SWE imaging on the bilateral fingers and hands. Additional ultrasound evaluation was carried out in 13 patients (9 dcSSc and 4 lcSSc) on their dorsal forearms, followed by skin biopsy conducted in the same skin areas. Correlations between ultrasound measurements and histological skin thickness and modified Rodnan skin score (mRSS) were investigated using Spearman’s correlation.

**Results:**

Compared with controls, ultrasound-measured skin thickness and skin stiffness were significantly higher in patients with SSc (*p* < 0.001) and even higher in those with dcSSc. No clear correlation could be established between ultrasound-determined skin thickness and stiffness at the same site. Ultrasound-measured skin thickness correlated well with histological skin thickness (*r* = 0.6926, *p* = 0.009). A weaker association was also observed between histological skin thickness and local mRSS (*r* = 0.5867, *p* = 0.050).

**Conclusions:**

Ultrasound is a reliable tool for quantifying skin involvement in SSc. Ultrasound-measured skin thickness showed good agreement with histological skin thickness.

## Background

Systemic sclerosis (SSc) is an autoimmune disease characterized by progressive fibrosis affecting the skin and internal organs [1]. Skin sclerosis is almost a universal manifestation of SSc, with more than 90% of patients being affected, leading to decreased mobility, flexion contracture, and ultimately severe functional impairment [1, 2]. Moreover, the extent of skin involvement is a predictive marker of survival, lung function, and heart involvement, while improvement in the skin is associated with better prognosis [3–5].

Modified Rodnan skin score (mRSS), which assesses skin thickness based on palpation at 17 body sites, is commonly used to semi-quantify the extent and severity of skin involvement in SSc [6]. The skin score has been proved to be well correlated with the histological extent of skin fibrosis, and thus being widely adopted in both routine care and clinical trials [7, 8]. Despite its good validity, certain defects still exist, including substantial inter-observer variability, relatively low sensitivity to subtle changes, probably poor clinical trial discrimination, and incompetence in distinguishing between thickened, hardened, and tethered skin [9, 10].

Skin ultrasound has been recognized as a valuable supplement to clinical skin assessment in SSc. First, subtle skin thickening was detectable by ultrasound even in the skin areas with a normal mRSS [10, 11]. Second, ultrasound was more sensitive to treatment-induced skin changes than clinical skin scores [12]. Third, ultrasound assessment of dermal thickness showed excellent repeatability within and across examiners [13]. Moreover, with the help of shear wave elastography (SWE), a novel imaging technique, ultrasound is capable of quantifying skin fibrosis in SSc [14]. However, there is a paucity of data on criterion validity of skin ultrasound in SSc. Besides, the relationship of ultrasound-measured skin thickness and stiffness with clinical skin score has not been fully explored.

Hence, the aims of the study were to further investigate the feasibility and reliability of ultrasound-based skin thickness and stiffness evaluation in SSc and, more importantly, to validate ultrasound measures against skin histology.

## Methods

### Study population

From June 2018 to May 2019, 44 consecutive SSc patients were enrolled from Huashan Hospital (Fudan University, China). All patients fulfilled the 2013 American College of Rheumatology/European League Against Rheumatism classification criteria [15] and were divided into diffuse cutaneous SSc (dcSSc) and limited cutaneous SSc (lcSSc) according to the extent of skin involvement [16]. The same experienced rheumatologist (M.R.L.) performed mRSS assessment on all patients, where 0 = normal skin, 1 = slight thickening, 2 = moderate thickening, 3 = severe thickening at each site, and the scores for all 17 sites were summed up to achieve a total score. Disease duration was calculated from the onset of the first non-Raynaud’s phenomenon symptom. Twenty-two age- and gender-matched healthy individuals were recruited as the control group. Ultrasound assessment of skin thickness and stiffness was performed in all participants on the fingers and hands. Skin biopsy, as well as synchronous ultrasound evaluation, was performed in 13 SSc patients at the identical sites on their dorsal forearms. Informed consent was obtained from every participant. The study was approved by the Ethics Committee of Huashan Hospital, Fudan University.

### Ultrasound examination

**Fig. 1:**
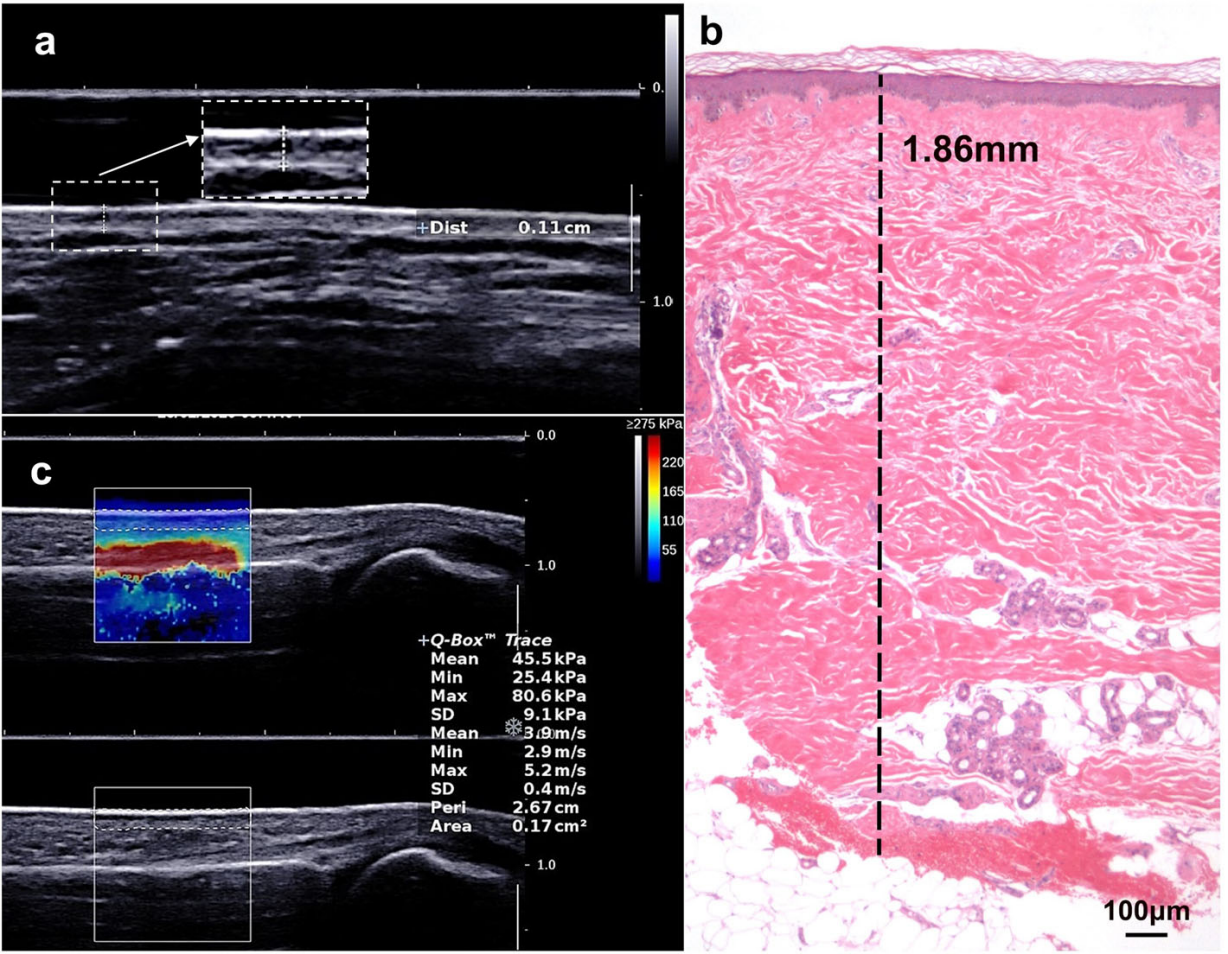
Representative ultrasonic and histological skin images of a patient with systemic sclerosis. In an ultrasound B-mode image (**a**) or a hematoxylin and eosin-stained image (**b**), the distance from the outermost epidermis to the interface between the dermis and subcutis was measured as skin thickness. Representative measurements were shown by dotted lines. To quantify skin stiffness, shear wave elastography mode was activated and a color-coded elastogram was superimposed on the B-mode image; the epidermis and dermis layers were traced, and the mean elastic modulus value of the trace area (the dotted box) was automatically calculated by the ultrasound system (**c**)

Skin thickness and stiffness evaluation was performed using the Aixplorer® ultrasound system (Supersonic Imagine, France) equipped with a 4–15-MHz linear probe. Measurements were made on 4 sites: right and left middle fingers (dorsum of the proximal phalanx) and right and left hands (dorsum of the index/middle metacarpal interspace, 2 cm proximal to the metacarpophalangeal joints). Extra measurements were conducted on the dorsum of unilateral forearms in patients who would undergo forearm skin biopsy. The ultrasound probe was placed at the junction of the distal one third and proximal two thirds of the forearm, exactly where biopsy would be performed later on. During the whole assessment process, the probe was maintained perpendicular to the skin without pressure. Total skin thickness (TST) was defined as the distance from the outermost epidermis to the interface between the dermis and the subcutis on B-mode imaging (Fig. 1a). To quantify skin stiffness, SWE mode was activated and a color-coded elastogram was superimposed on the B-mode image as the region of interest (ROI). The color represented the elastic modulus, and higher elastic modulus reflected higher tissue stiffness. The measurement scale was adjusted as needed with a maximum of 200–600 kPa. When stable color was obtained, the epidermis and dermis area in ROI was carefully traced. Total skin elasticity (TSE) was defined as the mean elastic modulus value of the trace area, which was calculated automatically by the ultrasound system (Fig. 1b). All measurements were made in triplicate and averaged. For intra-observer variability assessment, all 44 SSc patients were examined twice by the same sonographer (Y.C.) on two occasions, 2 to 4 h apart. For inter-observer variability assessment, 20 patients with SSc were examined by a second sonographer (Y.H.C.) on the same day. All ultrasound measurements were done blinded to the mRSS scoring.

### Skin biopsy

Excisional skin biopsy was conducted in 13 SSc patients on the dorsum of the unilateral forearm (at the junction of its distal one third and the proximal two thirds). We selected those patients with newly diagnosed and untreated SSc for skin biopsy. A fusiform skin sample, 5–10 mm in diameter, was incised down to the subcutaneous fat layer. The specimens were fixed in 10% buffered formalin, dehydrated and embedded in paraffin wax, and stained by hematoxylin and eosin (H&E). Histological images were captured under a light microscope (DMI4000B, Leica) and analyzed using ImageJ (version 1.52). The histological skin thickness was defined as the distance from the outermost epidermis to the interface between the dermis and subcutis (Fig. 1b). Measurements were performed in triplicate for every image and repeated in five consecutive images for every patient. The mean value of these repetitive measurements was adopted as the final result.

### Statistical analysis

Kruskal-Wallis *H* test with Dunn’s correction for multiple comparisons was used to analyze three or more groups. Relationships between variables were assessed using Spearman’s correlation. To evaluate the intra- and inter-observer reproducibility of ultrasound measurements, intraclass correlation coefficients (ICCs) were computed in single-measure, absolute-agreement, two-way mixed effects models. Statistical analyses were performed with SPSS Statistics (version 22.0), and graphs were constructed with GraphPad Prism (version 6.0). *p* < 0.05 were considered as statistically significant.

## Results

### Skin thickness and stiffness measured by ultrasound

**Fig. 2:**
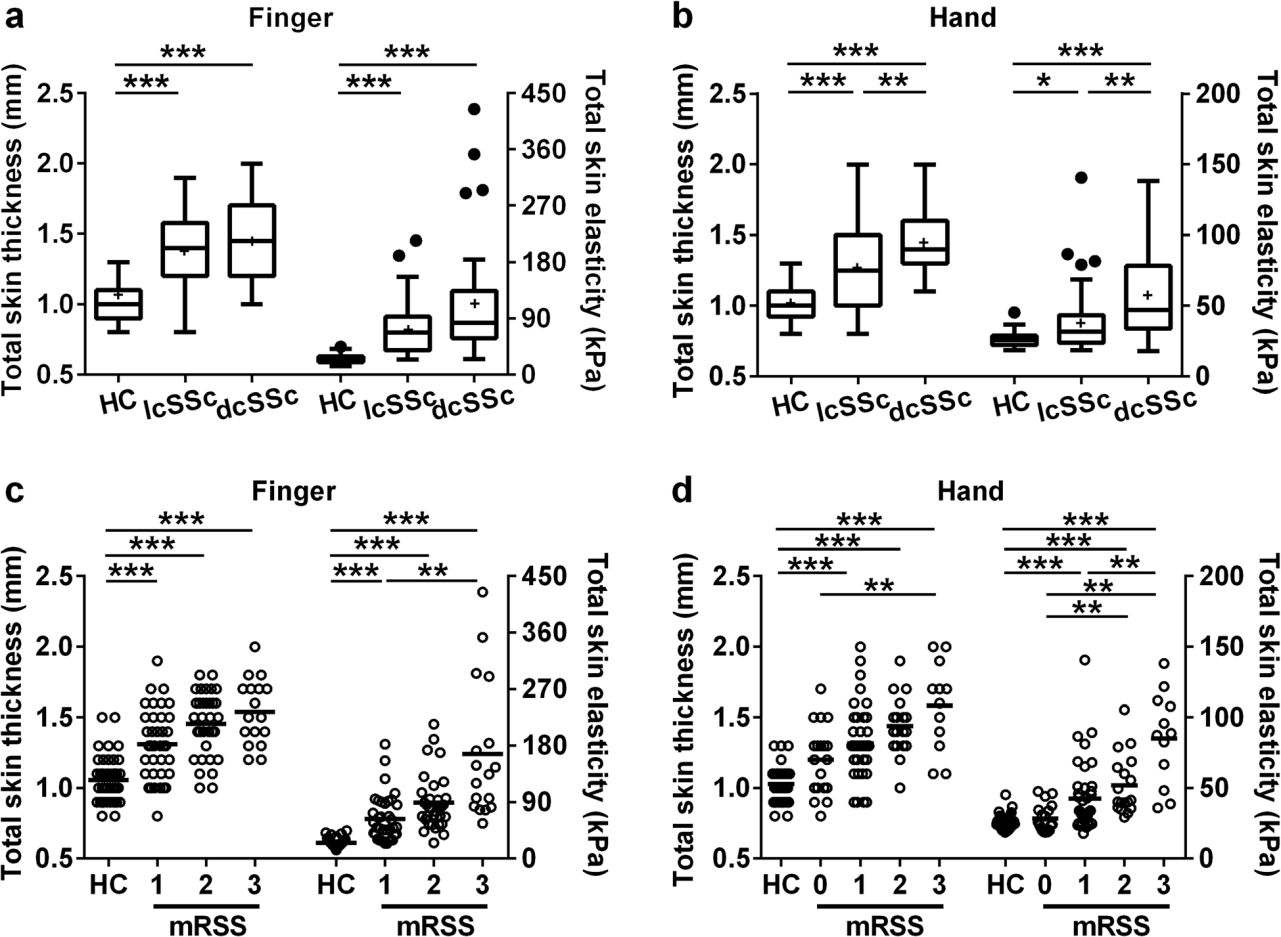
Ultrasound-measured skin thickness and stiffness stratified by disease subtype and modified Rodnan skin score. In box and whisker plots (**a**, **b**), lines from the bottom to the top indicated the minimum, 25th percentile, median, 75th percentile, and maximum values; dots indicated outliers, and crosses indicated mean values. In scatter plots (**c**, **d**), a single dot denoted a single measurement, and the dashes denoted the mean values. Kruskal-Wallis test with Dunn’s correction for multiple comparisons was conducted. Outliers were identified by the Turkey method. ***p < 0.001, **p < 0.01, *p < 0.05

Overall, 22 patients with dcSSc and 22 patients with lcSSc underwent ultrasound assessment. Clinical and ultrasonic features based on disease subtype were summarized in Table 1. Compared with healthy controls, patients with either dcSSc or lcSSc had higher skin thickness and stiffness at the fingers and hands. Among patients, skin thickness and stiffness did not differ significantly between the dcSSc and lcSSc subgroups at the fingers, whereas dcSSc patients showed remarkably higher skin thickness and stiffness at the hands than lcSSc patients, with a median TST of 1.40 mm (IQR, 1.30–1.60) and 1.25 mm (IQR, 1.00–1.50) (*p* = 0.004) and a median TSE of 46.9 kPa (IQR, 34.4–71.9) and 31.6 kPa (IQR, 24.0–41.7) (*p* = 0.001), respectively (Fig. 2a, b). In dcSSc, no clear correlation was observed between ultrasound-measured skin thickness and skin stiffness (*r* = 0.111, *p* = 0.474 at the fingers; *r* = 0.191, *p* = 0.215 at the hands), while a positive but very weak correlation was found in lcSSc (*r* = 0.348, *p* = 0.021 at the fingers; *r* = 0.334, *p* = 0.027 at the hands) (Suppl. Fig. S1 in Additional file 1).

**Table 1:** Clinical and ultrasonic features of SSc patients

Features	HC (*N* = 22)	lcSSc (*N* = 22)	dcSSc (*N* = 22)
Female, *n* (%)	15 (68.2)	16 (72.7)	14 (63.6)
Age, years	47.7 ± 11.7	50.6 ± 15.0	49.6 ± 11.7
Disease duration^a^, years	–	4.2 ± 3.7	2.6 ± 2.5
mRSS	–	6.0 ± 3.6	18.9 ± 9.6
TST at finger (IQR), mm	1.00 (0.90–1.10)	1.40 (1.20–1.52)	1.45 (1.20–1.70)
TST at hand (IQR), mm	1.00 (0.98–1.10)	1.25 (1.00–1.50)	1.40 (1.30–1.60)
TSE at finger (IQR), kPa	24.8 (20.3–28.8)	67.2 (39.1–92.0)	82.7 (58.5–131.5)
TSE at hand (IQR), kPa	25.7 (22.4–28.8)	31.6 (24.0–41.7)	46.9 (34.4–71.9)

*SSc* systemic sclerosis, *dcSSc* diffuse cutaneous SSc, *lcSSc* limited cutaneous SSc, *HC* healthy control, *mRSS* modified Rodnan skin score, *TST* total skin thickness, *TSE* total skin elasticity, *IQR* interquartile range

*Plus-minus values are means ± SD

^a^Disease duration was calculated from the onset of the first non-Raynaud’s phenomenon

### Correlations of ultrasound measurements and mRSS

In regions of clinically appreciable scleroderma (i.e., mRSS = 1, 2, or 3), both TST and TSE of SSc patients were significantly higher than those in the respective region of healthy controls. Ultrasound-measured thickness and stiffness tended to increase in accordance with the increase of local mRSS. Notably, both TST and TSE varied widely within each mRSS subgroup, while the same ultrasound values could be observed among different mRSS subgroups (Fig. 2c, d). We further explored the correlation between whole-body mRSS and ultrasound parameters measured at the fingers and hands; the results showed that whole-body mRSS tended to correlate closer to the finger or hand skin stiffness than thickness (Suppl. Fig. S2 in Additional file 1).

### Correlations of histological skin thickness and ultrasound measurements

**Fig. 3:**
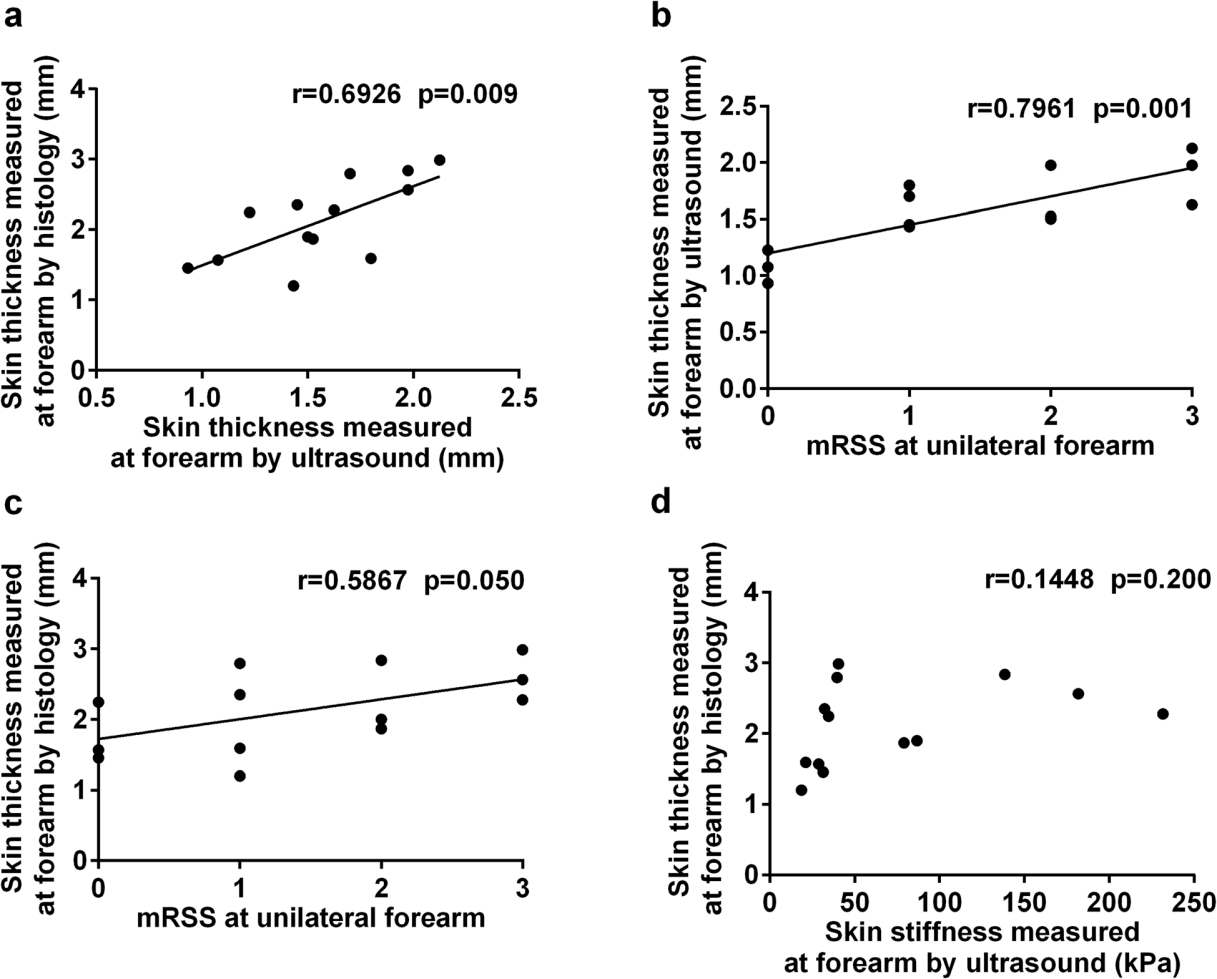
Correlations between ultrasound measurements and histological skin thickness and modified Rodnan skin score. Analyses were conducted using Spearman’s correlation

A total of 13 patients underwent forearm skin biopsy, of whom 9 (69.2%) were dcSSc and 4 (31.7%) were lcSSc. Patient demographics and clinical features are shown in Table 2. Forearm skin thickness measured by ultrasound correlated well with that measured by histology (*r* = 0.6926, *p* = 0.009) and by mRSS (*r* = 0.7961, *p* = 0.001) in the same region. Association was also observed, albeit weaker, between histological skin thickness and local mRSS (*r* = 0.5867, *p* = 0.050). No evident linear correlation was found between histological skin thickness and ultrasound-measured skin stiffness (*r* = 0.1448, *p* = 0.200) (Fig. 3).

**Table 2:** Clinical features of SSc patients undergoing skin biopsy

Features	*N* = 13
Gender (female/male)	7/6
Age (IQR), years	55 (51–57)
Disease subtype (dcSSc/lcSSc)	9/4
Disease duration^a^ (IQR), years	2 (0.8–5)
Local mRSS^b^ (0/1/2/3)	3/4/3/3
Total mRSS^c^ (IQR)	19 (7–24)
Antibody positivity (ATA/ACA/ARA)	6/2/0

*SSc* systemic sclerosis, *dcSSc* diffuse cutaneous SSc, *lcSSc* limited cutaneous SSc, *mRSS* modified Rodnan skin score, *IQR* interquartile range, *ATA* anti-topoisomerase I antibody, *ACA* anti-centromere antibody, *ARA* anti-RNA polymerase III antibody

^a^Disease duration was calculated from the onset of the first non-Raynaud’s phenomenon

^b^Local mRSS referred to the mRSS in the biopsy region (unilateral forearm)

^c^Total mRSS referred to the total mRSS assessed at 17 body sites

### Repeatability of ultrasound measurements

Intra- and inter-observer repeatabilities are summarized in Table 3. For skin thickness, ICC of intra- and inter-observer evaluations ranged from 0.910 to 0.920 and from 0.901 to 0.937, respectively. For skin stiffness, ICC of intra- and inter-observer evaluations ranged from 0.945 to 0.957 and from 0.840 to 0.897, respectively.

**Table 3:** Reproducibility of ultrasound measurements

Measurements	Intraclass correlation coefficient (95% confidential interval)	
	Intra-observer	Inter-observer
TST		
Finger	0.910 (0.865–0.940)	0.901 (0.821–0.947)
Hand	0.920 (0.880–0.947)	0.937 (0.883–0.966)
TSE		
Finger	0.945 (0.918–0.964)	0.897 (0.813–0.944)
Hand	0.957 (0.934–0.971)	0.840 (0.718–0.912)

*TST* total skin thickness, *TSE* total skin elasticity

## Discussion

The current study examined the validity and reliability of skin ultrasound in SSc. In particular, ultrasound-measured skin thickness was proved to be well correlated with histological thickness. Moreover, in both thickness and stiffness assessment, the intra-observer and inter-observer reproducibility were good, with ICCs reaching 0.9 and 0.8, respectively. On the whole, these findings support the use of skin ultrasound for SSc patients in clinical practice, and potentially in clinical trials.

Despite increasing evidence on ultrasound skin assessment in SSc, standardized methodology has not been established and heterogeneity still exists in terms of which skin layers to be examined [17]. Histologically speaking, not only the dermis but also the epidermis was found to be significantly thickened in clinically involved scleroderma skin [7, 18]. However, Moore et al. proposed that ultrasound assessment should be confined to the dermis, since the reproducibility of epidermal measurements alone was poor, with inter-observer ICC below 0.35 [13]. This was probably due to that substantial deviation could occur when assessing the thickness of the ultrathin epidermal layer alone. Given that the 4–15-MHz probe used in the current study may not be qualified enough to clearly discern the epidermis-dermis interface, we examined the total thickness of the epidermis and dermis to guarantee the repeatability of measurements.

Till now, skin ultrasound examination still lacks criterion validity. Previous studies established a correlation between ultrasound measurements and mRSS, but few made direct comparison with histology, the gold standard [19, 20]. Ihn et al. found skin thickening could be detected by ultrasound before histological alterations occurred; however, the authors failed to detail the conclusion [21]. To our knowledge, the current study is the first one in SSc that verify ultrasound skin thickness against skin biopsy thickness. The results suggested that ultrasound was probably a more accurate reflection of skin biopsy thickness than the clinical score.

In terms of sensitivity, prior reports have shown the advantage of ultrasound in detecting subclinical skin thickening which was not perceivable by physical examination [10, 11]. In the current study, we further revealed that patients with exactly the same mRSS could have markedly different ultrasound-measured skin thickness and skin stiffness. Collectively, the ability to discriminate subtle skin differences makes ultrasound a promising candidate for the early diagnosis and monitoring of SSc patients.

Stiffness assessment is another valuable but less frequently used outcome measure of skin ultrasound. Shear wave elastography showed the ability to differentiate the scleroderma skin from the normal skin in the present study, as in previous reports [22]. Inter-observer reproducibility was good with ICCs of 0.897 and 0.840 for fingers and hands, respectively, comparable to 0.975–0.989 and 0.700–0.857 reported before [23, 24]. Moreover, we found, compared with skin thickness, skin stiffness exhibited a stronger correlation with whole-body mRSS, suggesting that the latter might be a better disease severity-relevant parameter. Noteworthy, we reported for the first time that no clear correlation could be established between ultrasound-determined skin thickness and skin stiffness in SSc. It is a rather interesting finding, and the reason for the divergence probably lied in that skin thickening could result from interstitial edema or collagen deposition or both, leading to much perplexity, while skin stiffening reflected tissue fibrosis to a greater extent. Accordingly, skin thickness has been proved to be much higher in the edematous phase than in the fibrotic phase [20]. Given the high heterogeneity of SSc disease progression, concurrent ultrasound evaluation of skin thickness and stiffness may help clarify the underlying pathological conditions and help estimate the response to drug treatment of individuals.

Regarding feasibility, ultrasound with a probe frequency of 4–15 MHz is now readily available in clinical practice. Compared with biopsy, ultrasound assessment is a faster, safer, more cost-effective, and easy acceptable procedure. In our study, ultrasound measurement of thickness and stiffness took no longer than 3 min per site. Though it is still more time consuming than mRSS, ultrasound indeed provides a more comprehensive view of skin lesions, worth the extra time.

This study is limited by the small sample size and cross-sectional design. Longitudinal studies are needed to verify the responsiveness to change of ultrasound measurements against histological alterations. Another limitation is the low-frequency ultrasound used in our study which may hinder measurement accuracy and repeatability. Probe frequency at this level failed to offer high enough resolution images, so the epidermis-dermis interface was not readily discernable in all patients. As a result, we examined the total thickness of the epidermis and dermis instead of including only dermal measurements, while the latter was proposed to be more appropriate in evaluating scleroderma skin [13]. Moreover, those with dcSSc accounted for the majority who underwent skin biopsy in our center. This might be due to that invasive procedures were more acceptable to patients with progressive disease. Hence, it is necessary to explore the validity of skin ultrasound in various disease subtypes and in larger cohorts in the future.

## Conclusions

Our preliminary results shed new light on the use of skin ultrasound in SSc. We unveil for the first time a strong correlation between the ultrasound-measured skin thickness and the corresponding histological thickness. Besides, concurrent evaluation of thickness and stiffness by ultrasound may be a valuable supplement to clinical skin assessment in SSc.

## Supplementary information

**Additional file 1 **: **Supplementary Figure S1**. Correlations between ultrasound measured skin thickness and skin stiffness. **Supplementary Figure S2**. Correlations between whole body mRSS and ultrasound parameters measured at fingers and hands.

## Data Availability

The datasets used and/or analyzed during the current study are available from the corresponding authors on reasonable request.

## Abbreviations

SSc: Systemic sclerosis
dcSSc: Diffuse cutaneous systemic sclerosis
lcSSc: Limited cutaneous systemic sclerosis
mRSS: Modified Rodnan skin score
SWE: Shear wave elastography
TST: Total skin thickness
TSE: Total skin elasticity
ICCs: Intraclass correlation coefficients
