# Supplementary material for: Ultrasound assessment of skin thickness and stiffness: the correlation with histology and clinical score in systemic sclerosis
Source: Arthritis Res Ther. 2020 Aug 26;22:197. doi: 10.1186/s13075-020-02285-x (PMC7448329; doi:10.1186/s13075-020-02285-x)
Supplement: Supplementary file 1 — Additional file 1 : Supplementary Figure S1. Correlations between ultrasound measured skin thickness and skin stiffness. Supplementary Figure S2. Correlations between whole body mRSS and ultrasound parameters measured at fingers and hands. [file 13075_2020_2285_MOESM1_ESM.docx]

**Supplementary Figure S1. Correlations between ultrasound measured skin thickness and skin stiffness.**

**Supplementary Figure S2. Correlations between whole body mRSS and ultrasound parameters measured at fingers and hands.** Analyses were conducted using Spearman’s correlation.
